# Viscoelastic Damage Characteristics of Asphalt Mixtures Using Fractional Rheology

**DOI:** 10.3390/ma14195892

**Published:** 2021-10-08

**Authors:** Qipeng Zhang, Xingyu Gu, Zilu Yu, Jia Liang, Qiao Dong

**Affiliations:** 1 School of Transportation, Southeast University, Nanjing 211189, China; zhangqipeng@seu.edu.cn (Q.Z.); 220215104@seu.edu.cn (Z.Y.); liangjiahs@seu.edu.cn (J.L.); qiaodong@seu.edu.cn (Q.D.); 2 National Demonstration Center for Experimental Road and Traffic Engineering Education (Southeast University), Nanjing 211189, China

**Keywords:** pavement materials, asphalt mixture, compressive creep, damage evolution, fractional rheology theory, viscoelasticity

## Abstract

The mechanical behavior of asphalt mixtures at high stress levels are characterized by non-linear viscoelasticity and damage evolution. A nonlinear damage constitutive model considering the existence of creep hardening and creep damage mechanisms in the entire creep process is proposed in this study by adopting the fractional rheology theory to characterize the three-stage creep process of mixtures. A series of uniaxial compressive creep tests under various stresses were conducted at different temperatures to verify the model. The results indicated that the model predictions were in good agreement with the creep tests. The relationship between the model parameters and applied stresses was established, and the stress range in which the mixture exhibited only creep consolidation was obtained. The damage to the asphalt mixture was initiated in the steady stage; however, it developed in the tertiary stage. A two-parameter Weibull distribution function was used to describe the evolution between the damage values and damage strains at different stress levels and temperatures. The correlation coefficients were greater than 0.99 at different temperatures, indicating that a unified damage evolution model could be established. Thus, the parameters of the unified model were related to material properties and temperature, independent of the stress levels applied to the mixtures.

## 1. Introduction

Asphalt mixtures are considered as heterogeneous pavement materials typically composed of asphalt, aggregate, admixtures, and air voids. Furthermore, asphalt mixtures are the primary materials used worldwide for constructing pavement layers because of their remarkable advantages, such as short construction periods, long service lives, recyclability, ease of maintenance, and comfortable driving. Although aggregates, which are a type of rigid material, account for approximately 90% of all components within the asphalt mixtures, such mixtures exhibit complex rheological properties. Asphalt mixtures exhibit both viscous (fluid) and elastic (solid) behavior [1,2,3], and their mechanical properties are highly dependent on temperature, loading stress level, loading time, and loading speed [4,5]. The theoretical and experimental studies on the mechanical behaviors of asphalt mixtures provide a crucial basis for designing pavement layers [6,7]. Creep behavior is amongst the mechanical characteristics of asphalt mixtures [8], and it is strongly related to the rutting formation of the pavement structure [3,9]. Therefore, it is important to establish a constitutive model that can accurately predict the creep behavior of asphalt mixtures.

The improvement of constitutive models is gaining increasing interest in the field of rheology in order to better describe the mechanical behaviors of complex rheological materials. Furthermore, several rheological models of asphalt mixtures were proposed in the past [10,11,12,13,14,15,16,17,18,19]. These models can be divided into integral constitutive and differential constitutive models [15,20]. The integral viscoelastic constitutive model, which considers the loading history and memory of materials, is developed based on methods such as the Boltzmann superposition principle or the Leaderman modified superposition principle. Compared with multiple integral models, single integral models have fewer material parameters and simpler forms, which makes them easier to use in practical engineering. Schapery’s model, which is widely applied to characterize the viscoelastic properties of asphalt mixtures, is a single integral constitutive model [21]. Im et al. [22] described the viscoelastic and viscoplastic behavior of mixtures by combining Schapery’s viscoelastic model and Perzyna viscoplasticity with a generalized Drucker–Prager yield surface.

Classical integer differential constitutive models, such as the Maxwell model, Kelvin model, Burgers model, and generalized models, comprise springs and dashpots in series or in parallel. The mathematical expressions of the differential constitutive models can correspond to the mechanical models that have advantages of intuitionism and simplicity. The classical integer derivative constitutive models are widely used to analyze the creep behavior of asphalt mixtures. The Burgers model provides a better description of mechanical behavior than the Maxwell and Kelvin–Voigt models [23]. However, Cheng et al. [24] reported that, compared to the Burgers model, the generalized Kelvin (GK) model and the generalized Maxwell (GM) model have a better performance in describing the creep performance of asphalt mixtures. Although the classical constitutive models can describe the viscoelastic properties of asphalt mixtures, they yield a large error in describing the initial stage of the creep performance [13]. With the development of viscoelasticity theory, the fractional derivative operator that can describe the historical memory dependence and spatially wide relevance of a material meets the research requirements for examining the complex viscoelastic, non-Newtonian fluid and the porous media mechanics [25]. Furthermore, the fractional calculus theory achieves great success in describing the rheological behavior of asphalt materials [13,14,15,26,27]. Lagos-Varas et al. [13] established a viscoelastic model using fractional-order derivatives, and unlike the Burgers model, the fractional order model can predict the elastic jump observed at the beginning of the creep modulus. Celauro et al. [28] demonstrated that the fractional-order Burgers model can accurately characterize the creep/recovery response of asphalt mixtures. In addition, the fractional derivative model has the advantage of exhibiting a simpler expression, utilizing fewer parameters, and providing more accurate results. Furthermore analysis revealed that a fractional derivative model comprising two elements of fractional derivatives in series with a Maxwell element can be used to model the rheological behavior of asphalt binders. This includes the dynamic viscoelastic behavior, static creep, and relaxation characteristics [27].

The entire creep process of asphalt mixtures is characterized into three stages: primary (decelerated stage), steady (stationary stage), and tertiary (accelerated stage). Furthermore, different deformation mechanisms occur throughout the creep process of asphalt mixtures at various stresses. At a lower stress level, the asphalt mixture is gradually compacted and the aggregates are gradually stacked to form a stable skeleton structure, which results in a “consolidation effect” [19]. This deformation mechanism is known as the creep hardening mechanism [18], and the asphalt mortar resists deformation together with the skeleton structure during the procedure. However, at higher stress levels, the stress in the asphalt mixture exceeds the frictional resistance between the aggregates and the bonding between the aggregate and asphalt mortar, which results in the fragmentation of the asphalt mixture and the destruction of the skeleton. This consequently accelerates the viscoelastic flow rate and creep failure of the asphalt mixture in the tertiary stage. Here, the deformation mechanism is called the damage softening mechanism [18]. Linear models (e.g., the ordinary Burgers model, generalized Kelvin model, and fractional-order Burgers model) cannot describe the tertiary stage of creep.

One potential approach is to add a plastic element into the classic differential models to construct viscoelastoplastic models to accurately describe the three-stage creep behavior of asphalt mixtures at high stresses. The Nishihara model, which adds a plastic element to the Burgers model, was developed to describe the tertiary stage of creep and was used in the mechanical response analysis of bituminous materials [19]. A nonlinear viscoelastic-plastic creep model for asphalt mixtures was developed using variable-order fractional calculus, where time-varying viscoplastic elements were used to describe the tertiary stage of creep performance [15]. In addition, damage occurs in the asphalt mixture during the creep process. Therefore, another feasible approach is coupling a continuum damage evolution law with a linear viscoelastic model. Sun et al. [29] developed a damage constitutive model on the basis of the generalized Burgers model and the Perzyna viscoplastic flow theory, combined with a modified Rabotnov damage theory, which can better reflect the three stages of deformation in creep testing and the hardening and softening effects in the constant strain rate compression testing. Zhang et al. [18] suggested that the characteristics of the three-stage permanent deformation were attributed to a competition between the damage softening and strain hardening effect, and they proposed a viscoelastoplastic damage mechanics model wherein the damage and hardening variables were introduced to modify the Burger’s model for describing the deformation of asphalt mixtures.

Most existing creep damage models are formed by the integral order difference constitutive model coupled directly with the damage factor. These models have the disadvantage of many material parameters and overly complex expressions, which are not conducive to their generalization in practical engineering. Furthermore, these models directly couple the strain caused by damage (called the damage strain) to the viscoelastic strain of the asphalt mixture, which is not conducive to studying the strain development mechanism and damage characteristics during the creep process. Therefore, this study aims to propose a creep damage model based on the fractional derivative operator that considers both the creep hardening mechanism and damage softening mechanism to characterize and analyze the creep process of asphalt mixtures at various stress levels and temperatures. Simultaneously, a unified damage evolution model is constructed for different stress levels, and the development of the strain and damage characteristics of the asphalt mixture throughout the creep process is analyzed. Finally, a method for the statistical quantification of the damage evolution of the asphalt mixture is proposed.

The paper is structured in the following way: a nonlinear viscoelastic creep damage model adopting the fractional rheology theory is developed in Part 2. The compression creep tests are conducted on AC-13 asphalt mixtures in Part 3. In Part 4, the experimental results are described and the proposed model is experimentally validated. Meanwhile, the model parameters and the creep damage evolution of the asphalt mixtures are analyzed in Part 4. Finally, several conclusions are drawn in Part 5.

## 2. Theoretical Background

### 2.1. Modelling of Creep Hardening Mechanism

**Figure 1:**
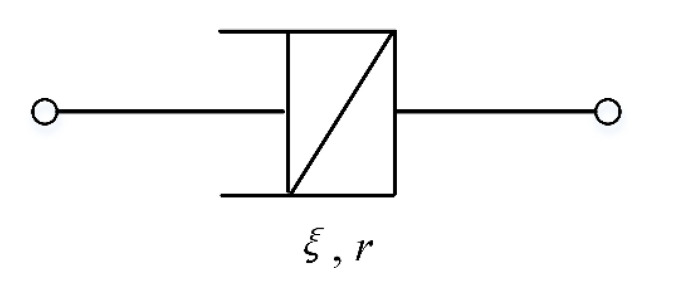
Schematic of fractional viscoelastic element (Abel spring-pot element).

In the classical integer-order differential viscoelastic model, the elastic mechanical behavior of the material is modeled with a Hook spring, and the viscous behavior is modeled with a Newton dashpot. The viscoelastic behavior of the asphalt mixtures is achieved by various combinations of Hook springs and Newton dashpots in series or in parallel. Gu et al. [26] adopted the Abel spring-pot element to describe the viscoelastic behavior based on the fractional order calculus theory for better describing the complex viscoelastic mechanical behavior of the materials. The fractional order viscoelastic element (Abel spring-pot element) is shown in Figure 1 and the stress–strain relation of the Abel spring-pot element is expressed as in [26]:

(1) $$\sigma \left(t\right)=\xi D^{r}\varepsilon \left(t\right)\left(0\leq r\leq 1\right),$$

where *σ*, *t*, *ε*, *ξ*, and *D^r^* denote the nominal stress, function variable, strain, material coefficient, and differential operator of the Riemann–Liouville fractional calculus [30], respectively, which is defined as:

(2) $$D^{r}f\left(t\right)=\frac{d^{r}f\left(t\right)}{dt^{r}}=\frac{1}{\Gamma \left(1-r\right)}\frac{d}{dt}\int_{0}^{t}\frac{f\left(\tau \right)}{{\left(t-\tau \right)}^{r}}d\tau \left(0\leq r\leq 1\right),$$

where *r* denotes the fractional order of differentiation, *Г*(*r*) denotes the gamma function, and $\Gamma \left(r\right)=\int_{0}^{\infty }t^{r-1}e^{-t}dt$. According to Equations (1) and (2), the spring-pot reduces to the Hook spring when *r* = 0, whereas it reduces to the Newton dashpot when *r* = 1. Thus, the spring-pot can be regarded as a type of fractional differential viscoelastic element.

The strain can be obtained from Equation (1) as:

(3) $$\varepsilon \left(t\right)=\frac{1}{\xi }D^{-r}\sigma \left(t\right),$$

where *D*^-*r*^ denotes the integral operator of the Riemann–Liouville fractional calculus [30], which is defined as:

(4) $$D^{-r}f\left(t\right)=\frac{d^{-r}f\left(t\right)}{dt^{-r}}=\int_{0}^{t}\frac{{\left(t-\tau \right)}^{r-1}}{\Gamma \left(r\right)}f\left(\tau \right)d\tau \left(0\leq r\leq 1\right),$$

In the case of the creep, under a constant stress level$\sigma \left(t\right)=\sigma_{0}H(t)$, the creep strain can be obtained from Equations (3) and (4) as:

(5) $$\varepsilon \left(t\right)=\frac{\sigma_{0}t^{r}}{\xi \Gamma \left(r+1\right)}=\frac{\sigma_{0}}{K}t^{r}\;\left(0\leq r\leq 1\right),$$

where *K* is related to the coefficient *ξ* and the fractional order *r* of the material, and$K=\xi \Gamma \left(r+1\right)$. *H*(*t*) denotes the Heaviside step function, that is, $H\left(t\right)=\left\{\begin{array}{cc} 1 & t>0\\ 0 & t\leq 0 \end{array}\right.$. According to Equation (5), *K* is related to the deformation of the material and referred to here as the deformation factor.

Find the derivative of Equation (5) with respect to *t*:

(6) $$\dot{\varepsilon }=\frac{d\varepsilon }{dt}=\frac{\sigma_{0}r}{K}t^{r-1}\left(0\leq r\leq 1\right).$$

**Figure 2:**
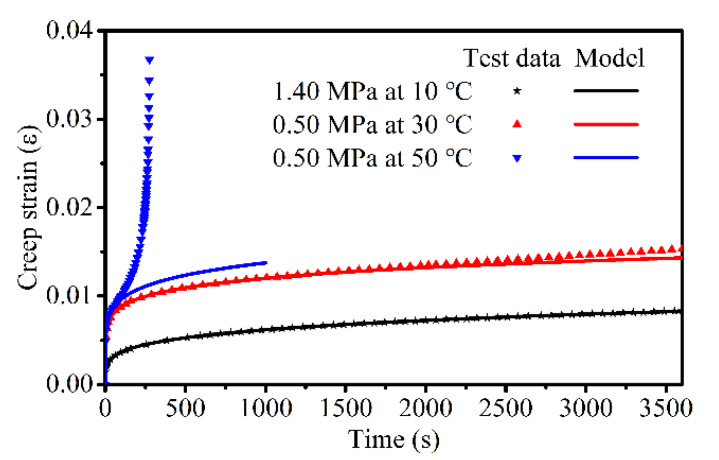
Consolidation effect and decelerating stage of the asphalt mixture: Abel model vs. test.

Equation (6) indicates that the strain rate of the material, which decreases as time increases, and is consistent with the decrease in the strain rate at the decelerating stage of creep. When time *t* is sufficiently large, the strain rate converges to zero, which is consistent with the consolidation effect at the lower stress levels [19]. Figure 2 shows the Abel model used to describe the consolidation effect and the decelerated stage of creep. (The test data in the figure were randomly selected from the creep tests in Section 3 to illustrate the applicability of the model.) Figure 2 shows that the Abel model can characterize both the hardening effect and the primary stage of creep performance very effectively. Thus, the Abel spring-pot model can be applied to characterize the creep hardening mechanism of asphalt mixtures.

The load is kept constant ($\sigma \left(t\right)=\sigma_{0}H(t)$) for an assigned time$\bar{t}$, where the viscoelastic material enters the creep phase. When $t>\bar{t}$, the load is removed and the material enters the recovery phase, and the stress is zero. The stress in the creep recovery phase can be expressed as:

(7) $$\sigma \left(t\right)=\sigma_{0}H\left(t\right)-\sigma_{0}H\left(t-\bar{t}\right).$$

where $\bar{t}$ denotes the loading time.

The creep-recovery strain can be obtained from Equations (5) and (7) as:

(8) $$\varepsilon \left(t\right)=\frac{\sigma_{0}}{Κ}\left\{t^{r}-H\left(t-\bar{t}\right)\cdot {\left|t-\bar{t}\right|}^{r}\right\}\;\left(0\leq r\leq 1\right).$$

Equation (8) shows that for aviscoelastic materials, there are always irrecoverable strains in the material, no matter how much stress is applied. In other words, viscoelastic materials have permanent strains at any time after being stressed.

### 2.2. Modeling of Damage Softening Mechanism

There is a damage effect throughout the creep of the asphalt mixture, and the damage evolution results in a nonlinear mechanical behavior. Herein, the damage variable *D* is used to describe the deterioration of the Abel spring-pot element, and damage evolution is related to the stress levels and its own distributed microdefects in the asphalt mixture. Based on the Kachanov damage theory [31], the damage evolution can be defined as:

(9) $$\frac{dD}{dt}=C\sigma^{q}{\left(1-D\right)}^{-v},$$

where *C*, *u*, and *v* represent the temperature-dependent material parameters, and 0≤D≤1. 

Taking the integral of Equation (9) with the initial condition *D* = 0 at *t* = 0+ and the critical condition *D* = 1 at *t* = *t_f_* yields the damage evolution as [19]:

(10) $$D=1-{\left[1-\left(1+v\right)\int_{0}^{t}C\sigma^{u}dt\right]}^{1/\left(1+v\right)}\;\left(0\leq t\leq t_{f}\right),$$

where *t_f_* denotes the damage-induced failure time. 

For $\sigma \left(t\right)=\sigma_{0}H(t)$, the damage evolution of creep can be written as:

(11) $$D=1-{\left(1-t/t_{f}\right)}^{1/\left(v+1\right)},$$

where *t_f_* can be expressed as:

(12) $$t_{f}=\frac{1}{C(1+v)\sigma_{0}^{u}},$$

According to the principle of the strain equivalence of the continuum damage mechanics [32], a constitutive relationship of the damaged material is obtained by replacing the nominal stressσwith the effective stress$\bar{\sigma }$. The relationship betweenσand$\bar{\sigma }$is expressed as:

(13) $$\bar{\sigma }=\frac{\sigma }{1-D},$$

Combining Equations (5), (11), and (13), the creep strain, considering the damage evolution based on the fractional derivative operator, can be expressed as:

(14) $$\varepsilon \left(t\right)=\frac{\sigma_{0}}{K\cdot \left(1-D\right)}\cdot t^{r}=\frac{\sigma_{0}t^{r}}{K}\cdot {\left(1-t/t_{f}\right)}^{-1/\left(v+1\right)}=\frac{\sigma_{0}}{K^{'}}\cdot t^{r}\left(0\leq r\leq 1\right),$$

where$K^{'}=K\cdot \left(1-D\right)$. As can be seen from Equation (14), the damage causes the deformation coefficient to no longer be constant, and as the damage accumulates, the deformation coefficient deteriorates and the strain increases until the specimen is damaged or fails.

In this study, the creep strain, when considering the damage, is decomposed into a strain caused by the damage and the strain from the creep hardening mechanism in the undamaged state. The strain induced by the damage (referred to as the damage strain in this study) can be expressed as:

(15) $$\varepsilon_{d}\left(t\right)=\frac{\sigma_{0}t^{r}}{K\left(1-D\right)}-\frac{\sigma_{0}t^{r}}{K}=\left(\frac{1}{1-D}-1\right)\;\cdot \frac{\sigma_{0}t^{r}}{K}\left(0\leq r\leq 1\right),$$

where *ε_d_* denotes the damage strain, and this can be applied to characterize the damage softening mechanism of the asphalt mixtures.

### 2.3. Modeling of Fractional Derivative Creep Damage Model

**Figure 3:**
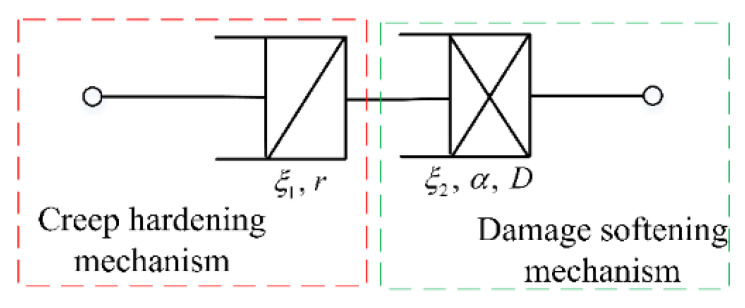
Schematic of fractional creep damage model.

A simple creep damage model is proposed by combining a fractional derivative model that describes the creep hardening mechanism with a fractional derivative damage strain that describes the damage deterioration mechanism, as shown in Figure 3. Luo et al. [33] described the asymmetry of the dynamic viscoelastic properties of asphalt mixtures using a modified fractional Zener model, where the two spring-pot elements have different fractional orders *r* and the same material coefficient *ξ*. In this study, it is suggested that not only should the two spring-pot elements describing the two mechanisms have different *r*, the coefficients *ξ* should also not be the same to better study the creep performance of asphalt mixtures. The fractional derivative creep damage model can then be expressed as:

(16) $$\varepsilon \left(t\right)=\frac{\sigma_{0}t^{r}}{K_{1}}+\frac{\sigma_{0}t^{\alpha }}{K_{2}}\left[{\left(1-t/t_{f}\right)}^{-1/\left(v+1\right)}-1\right],$$

where *r* and *α* denote the fractional orders of the two spring-pot elements, and *ξ*_1_ and *ξ*_2_ denote the coefficients of the two spring pots.

## 3. Uniaxial Compression Static Creep Test

### 3.1. Material

**Figure 4:**
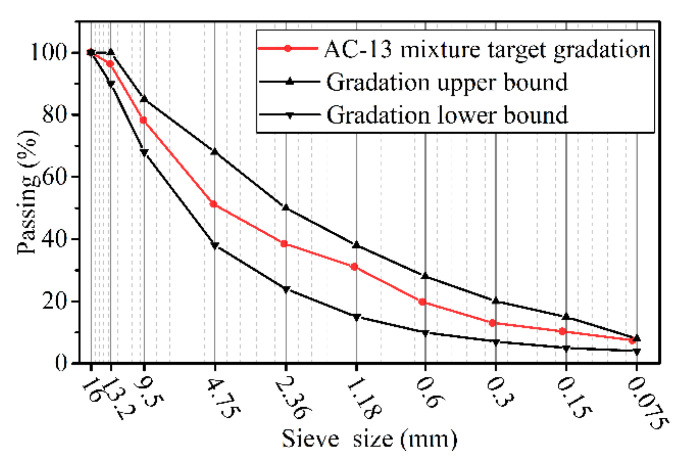
Gradations of the AC-13 asphalt mixture.

An AH-70 virgin binder was employed to form the AC-13 asphalt mixture, which contained basalt aggregates. The gradation of the asphalt mixture was designed according to the Standard Test Methods of Bitumen and Bituminous Mixtures for Highway Engineering (JTG E20-2011), as shown in Figure 4. The performance tests of the asphalt binder and basalt aggregate were carried out according to JTG E20-2011 and JTG E42-2005 (Test Methods of Aggregate for Highway Engineering), respectively. The performances of the asphalt binder and basalt aggregates are presented in Table 1 and Table 2. The optimum asphalt–aggregate mass ratio was determined to be 4.9% according to JTG E20-2011; the air voids in this study were set to 4.0 ± 0.2%. The Saint Venant principle clearly indicated that the larger the ratio of the height to the diameter of the specimen subjected to uniaxial stress, the greater the area beyond the end of the specimen that approached the uniaxial state. Therefore, this study adopted a gyratory compaction method to prepare a specimen with a height to diameter ratio of 1.5 for attenuating the effect of end friction on the stress state of the specimen. First, a certain amount of the mixture was compacted into a cylindrical specimen (diameter = 150 mm, height = 170 mm) using a gyratory compactor. The amount of asphalt mixture required to form the cylindrical specimen was calculated based on the maximum theoretical density, void ratio and specimen volume, and the amount should be such that the height of the specimen formed reached plus or minus 3 mm of the desired height. In this study, the amount of the mixture required to form a specimen was 7694 g. The parameters of the gyratory compactor were set according to the Superpave design method, where the internal rotation angle is 1.16° ± 0.02°, the vertical pressure was 600 ± 18 kPa, and the rotation rate was 30 r/min ± 0.5 r/min. Meanwhile, this study chose to set the required number of rotational compaction (100) as the end condition of rotational compaction. After compaction, the mold was demolded to obtain the specimen. Then, the formed specimen was core drilled, and the ends of the specimen were removed to obtain a cylindrical specimen (diameter = 100 mm, height = 150 mm).

### 3.2. Methodology

Creep tests were conducted on AC-13 asphalt mixtures at various stress levels and temperatures to comprehensively analyze the viscoelastic damage behavior of asphalt mixes. Various researchers selected different test temperatures and test stress levels. Ghorbani et al. [7,9] assessed the temperature effects of mixtures at 5, 20, 35, and 50 °C. Cheng et al. [24] analyzed the viscoelastic properties of different mixtures from 10 to 50 °C at 10 °C increments. Luo et al. [15] studied the viscoelastic properties of asphalt mixtures at a room temperature of 25 °C only. This study was based on the work of Cheng et al. [24] who analyzed the creep properties of the AC-13 asphalt mixture from 10 to 50 °C at 20 °C increments. Considering the temperature, the loading capacity of the instrument, and the loading time, the stress levels selected at different temperatures were different for exhibiting both the consolidation effect and the tertiary stage of creep performance at different temperatures. For example, at 50 °C and 0.8 MPa, the asphalt mixture exhibited the three stages of creep, while, at 10 °C, the asphalt mixture only exhibited a consolidation effect at 0.8 MPa. To allow the asphalt mixture to exhibit the three stages of creep, it required a larger stress level. Therefore, in this study, in order to study the change of viscoelasticity under different stresses, and to consider the comparison of viscoelasticity under the same stress at different temperatures, the creep test conditions shown in Table 3 were selected.

The specimen was kept under the testing temperature for more than 4 h before the experiment to ensure a consistent temperature throughout the specimen and to eliminate the effects of temperature inhomogeneity. Before the creep test, the specimen was preloaded with 0.05 MPa and held for 60 s to eliminate the effect of mechanical errors. Double-greased membranes was used to reduce the effect of friction between the clamps and the specimen. All tests were carried out with the UTM-25 tester (IPC Australia), which could apply a maximum stress of 2.8 MPa to the specimens. All preset stress levels were maintained for 3600 s at 30 and 50 °C. However, at 10 °C, the stress duration was increased to 10,000 s to ensure that the specimen exhibited the tertiary stage of creep at 2.8 and 2.5 MPa.

## 4. Test Results and Analysis

### 4.1. Test Results

**Figure 5:**
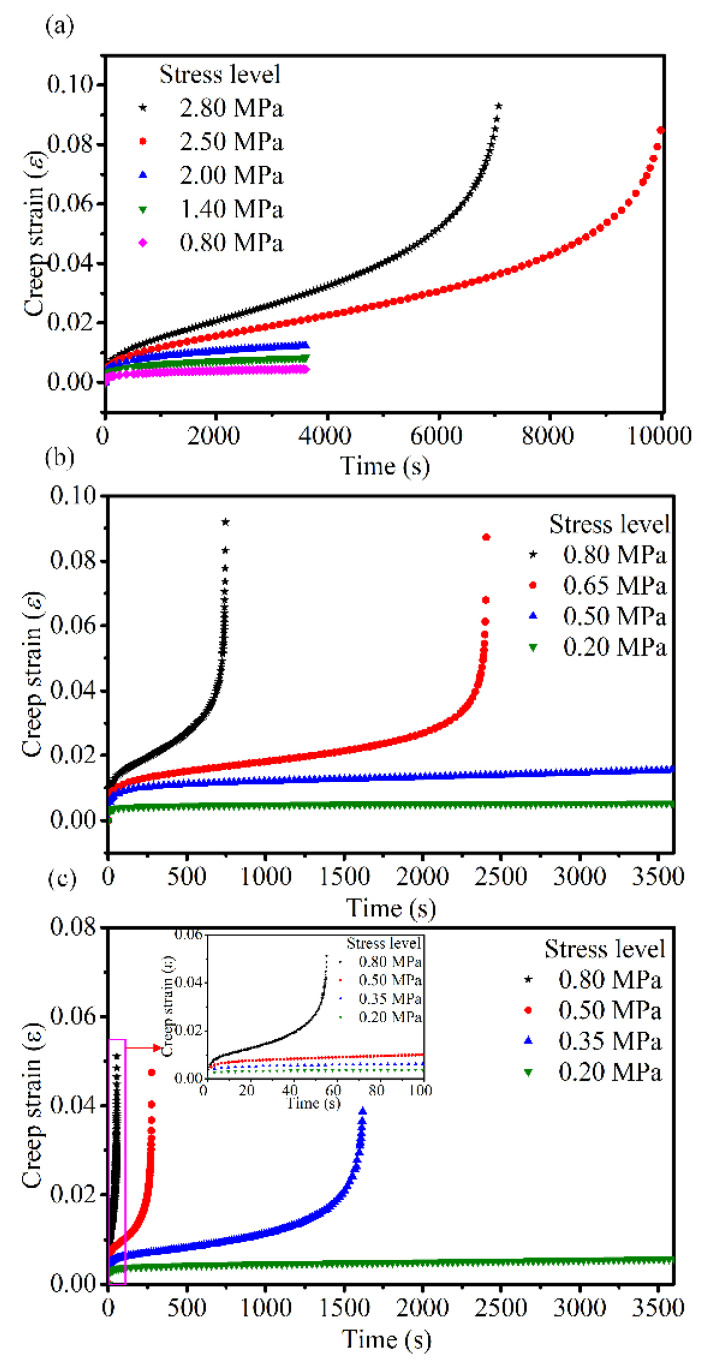
Creep strain curves of asphalt mixtures under different stress levels at (**a**) 10 °C, (**b**) 30 °C, and (**c**) 50 °C.

Three tests were performed at each stress level, and the results were averaged. The averaged creep strain vs. time curves for the applied stress levels at different temperatures are shown in Figure 5. 

**Figure 6:**
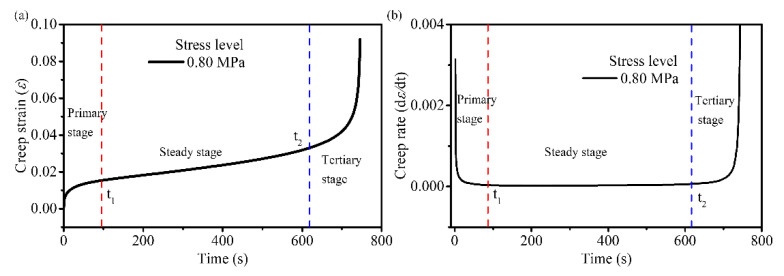
Schematic of the three stages (**a**) curve of three stages of creep, (**b**) curve of creep rate of asphalt mixture.

Figure 5 shows that the creep deformation of the asphalt mixtures is stress- and temperature-dependent. Here, the creep curve at 30 °C and 0.8 MPa is used as an example illustrate the three stages of creep and the time at which each stage begins. The curve of three stages of creep and the curve of creep rate are shown in Figure 6a,b, respectively. The creep rate curve was obtained by deriving the creep curve versus time. In Figure 6, *t*_1_ and *t*_2_ represent the start times of the steady stage and tertiary stage, respectively. Additionally, during this time, the creep rate remains almost constant and the value reaches a minimum. Therefore, the change in creep rate can be used to determine which creep stage the asphalt mixture is in. Combining Figure 5 and Figure 6, at all temperatures, the creep process under larger stress levels exhibited all three stages of creep, whereas under smaller stress levels it only exhibited decelerated and stationary stages (i.e., consolidation effects). During the primary stage, the strain rate of the asphalt mixture gradually decreased with increasing in loading time; this is referred to as creep hardening. Creep hardening is different from strain hardening, which refers to the phenomenon wherein the material enters plastic deformation and the strength increases with an increase in deformation. The prerequisite for strain hardening is that material stresses should reach the yield strength and produce irreversible plastic deformation, i.e., the hardening is attributed to the strengthening of the yield strength. However, even under lower stress, the asphalt mixture exhibited varying degrees of decay creep; thus, creep hardening in the decelerating stage was not attributed to strain hardening, but to the compaction effect of the asphalt mixture under compressive stress. In this stage, the asphalt mixture was gradually compacted under the compressive stress, the voids were reduced and the strength gradually increased. In the accelerated stage, a rapid increase in deformation in addition to an increase in the strain rate with time was observed because of the presence and evolution of the damage, and this eventually led to a creep failure in the asphalt mixture. The strain rate at the accelerated creep stage was related to the stress and temperature: the higher the stress, the greater the strain rate; the higher the temperature, the greater the strain rate.

Thus, based on the testing results of the creep performance, this study considers that there are two mechanisms in the entire creep process based on the creep testing results: creep hardening and creep damage deterioration. When the creep hardening mechanism is dominant, the strain rate of the asphalt mixture gradually decreases with an increase in loading time, and the creep decays. When the damage deterioration mechanism is dominant, the strain rate increases with an increase in loading time, and the creep exhibits an accelerated state. When the two mechanisms occur in proximity, the strain rate remains constant and asphalt mixture is in a stable state. The point at which the steady stage enters the accelerated stage is called the flow time. The flow time depends on the level of stress applied; the higher the stress level, the earlier the damage deterioration mechanism occurs, and thus the flow time is shorter.

### 4.2. Model Verification and Parameter Determination

**Figure 7:**
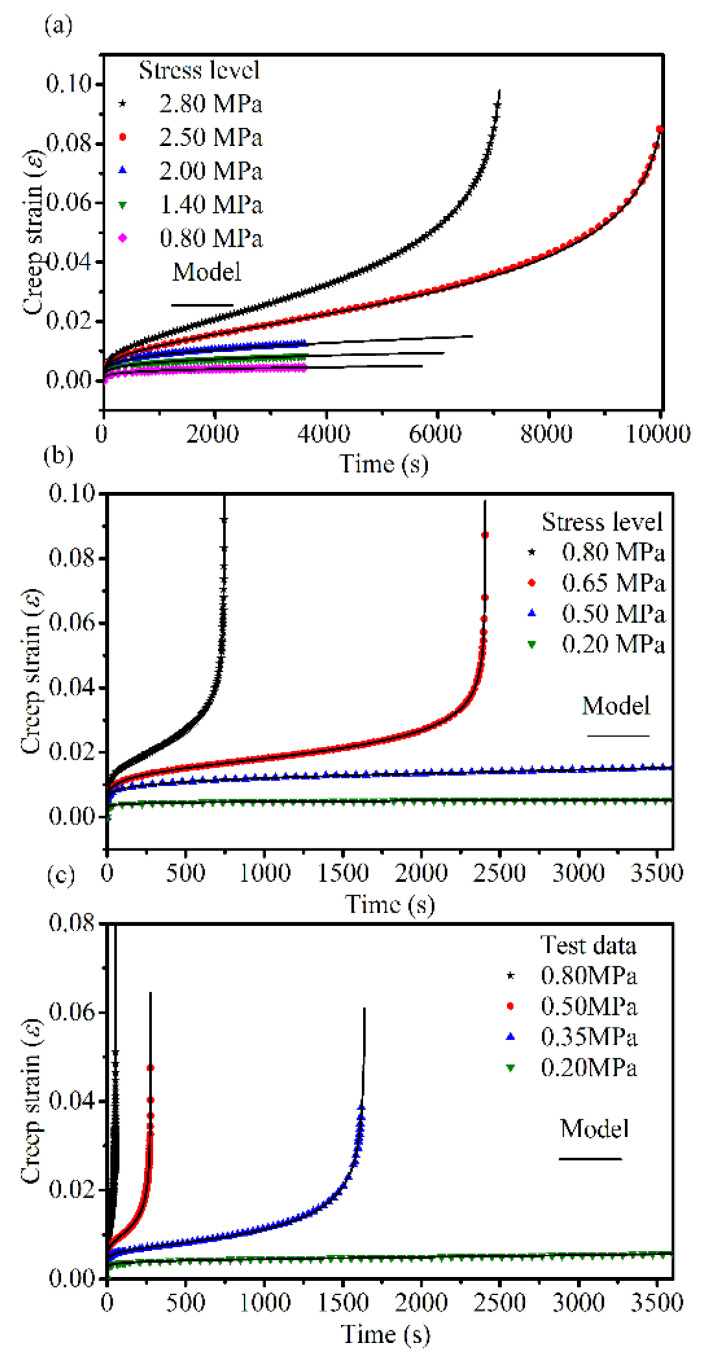
Comparison between the tests data of the creep tests and the fractional derivative creep damage model predicted creep curves at (**a**) 10 °C, (**b**) 30 °C, and (**c**) 50 °C.

We verify whether the proposed creep damage model can accurately describe and reproduce all creep behavior presented in the tests. Equation (16) is determined by a curve fitting to the creep test data using the Levenberg–Marquardt optimization algorithm in 1stop. The parameters of the proposed model are listed in Table 4; the testing data of creep tests under various conditions and the simulation results of the proposed model are illustrated in Figure 7. The parameters of the correlation coefficients R^2^ are greater than 0.998, which indicates that the fractional derivative creep damage model can accurately characterize the three-stage creep process of asphalt mixtures under various conditions.

Table 4 indicates that, at lower temperatures (10 °C), when the asphalt mixtures are subjected to smaller stress levels (0.8–2.0 MPa), the deformation factor *K*_1_, which describes the creep hardening mechanism, remains approximately constant and its value is much greater than at the other temperatures. This indicates that the deformation factor *K*_1_ is independent of the stress levels at lower stress levels (0.8–2.0 MPa) and the asphalt mixture has greater strength at lower temperatures (10 °C). This is most likely due to the fact that asphalt mortars have a greater strength and exhibit elastic behavior at lower temperatures. Additionally, in the smaller stress range, the asphalt mixture has a smaller strain, and the compaction within the asphalt mixture is not sufficient to change the skeletal structural strength of the mixture, so the deformation factor *K*_1_ remains constant. As only consolidation effects are exhibited within the asphalt mix at these small stresses, these smaller stress ranges (0.8–2.0 MPa at 10 °C and <0.2 MPa at 30 °C) are referred to as creep consolidation stress (CCT) ranges. The creep consolidation stress range is temperature-dependent. At higher temperatures (30 °C) the asphalt mixtures exhibit a creep consolidation only at a smaller stress level (0.2 MPa); while, at lower temperatures (10 °C), the asphalt mixtures exhibit creep consolidation behavior over a wide stress range (0.8–2.0 MPa).

In the creep consolidation state, the fractional order *r* increases as the stress increases and the parameters (*α*, *K*2), describing the damage mechanism, are close to zero. It follows from Equation (16) that when the deformation factor *K*_2_ tends to be zero, the damage must be sufficiently small to enable the proposed constitutive model to describe the creep properties. The strain induced by the damage is too small relative to the strain of the hardening mechanism. Therefore, the Abel model can be applied to characterize the entire creep process of an asphalt mixture under smaller stress levels. This once again shows that the Abel model can describe the consolidation effect of the asphalt mixes. Under other creep conditions, both the fractional order *r* and deformation factor *K*_1_ increase as the stress increases for the hardening mechanism. The fractional order increases because the flow rate of the asphalt mortar in the asphalt mixture increases as the stress increases, which causes the asphalt mixture to gradually exhibit a viscous behavior. The increase in the deformation factor indicates that the resistance to deformation increases with the increasing stress. As the stress increases in the hardening mechanism, the faster the asphalt mixture is compacted, the faster the increase in the skeletal strength between the aggregates, and the smaller the deformation. For the softening mechanism, the fractional order α increases with the increasing stress, whereas the deformation factor *K*_2_ decreases with the increasing stress. The fractional order increases because the flow rate of the asphalt mortar increases as the stress increases. The decrease in the deformation factor is attributed to the presence of damage in the softening mechanism; the greater the damage to the inter-aggregate asphalt mortar as the stress increases, and the greater the deformation. For the same stress level, asphalt mixtures exhibit different viscoelastic properties at different temperatures. For example, *r* increases and *K*_1_ decreases as the temperature increases for the stress of 0.8 MPa. This is because, at higher temperatures, the asphalt mortar exhibits a stronger viscosity, while at lower temperatures the asphalt exhibits a stronger elasticity. When the model is applied to describe the three stages of creep, i.e., when the hardening and deterioration mechanisms are present, the fractional order of the damage softening mechanism is larger compared to the hardening mechanisms at higher temperatures (30 °C and 50 °C). This is due to the presence of damage, which makes the asphalt mixture exhibit stronger viscosity. The deformation factor of the damage softening mechanism is also larger compared to the hardening mechanism. This is because the asphalt mixture is compacted in the primary stage, which makes its resistance to deformation increase when it enters the softening mechanism. However, at 10 °C, the parameter α describing the damage mechanism is small, indicating that the damage of the asphalt mixture at this temperature tends to occur more closely to the elastic damage.

### 4.3. Analysis of the Relationship between Model Parameters and Stress Levels

**Figure 8:**
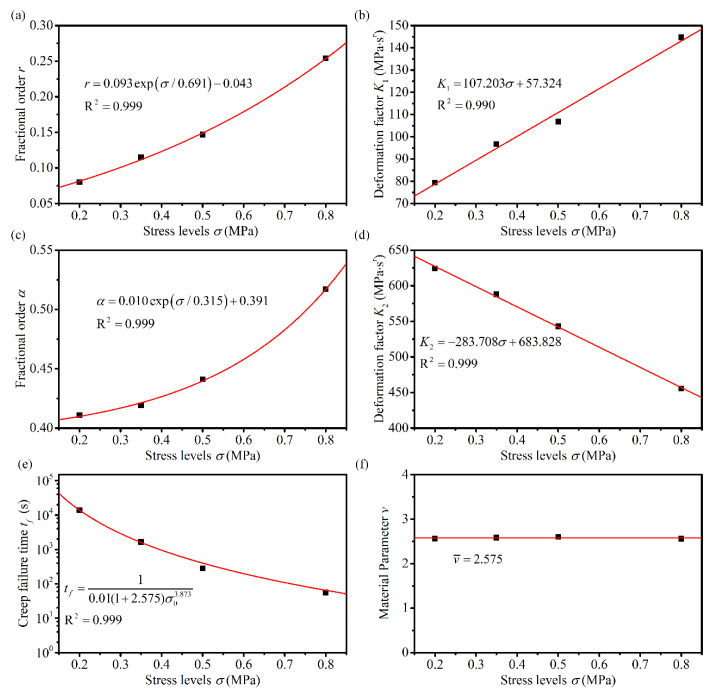
Variation of the proposed model parameters with stress levels at 50 °C (**a**) *r*, (**b**) *K*_1_, (**c**) *α*, (**d**) *K*_2_, (**e**) *t_f_*, and (**f**) *v*.

**Figure 9:**
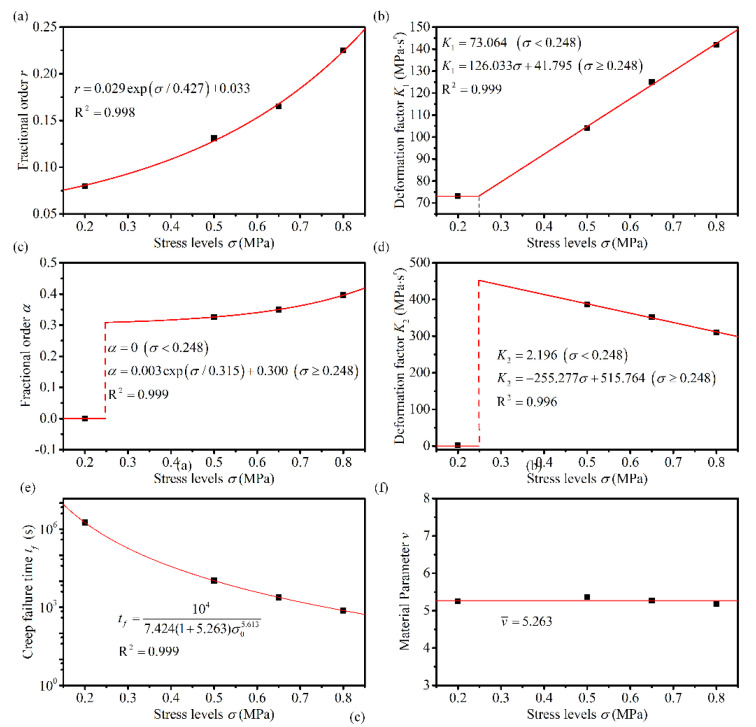
Variation of the proposed model parameters with stress levels at 30 °C (**a**) *r*, (**b**) *K*_1_, (**c**) *α*, (**d**) *K*_2_, (**e**) *t_f_*, and (**f**) *v*.

**Figure 10:**
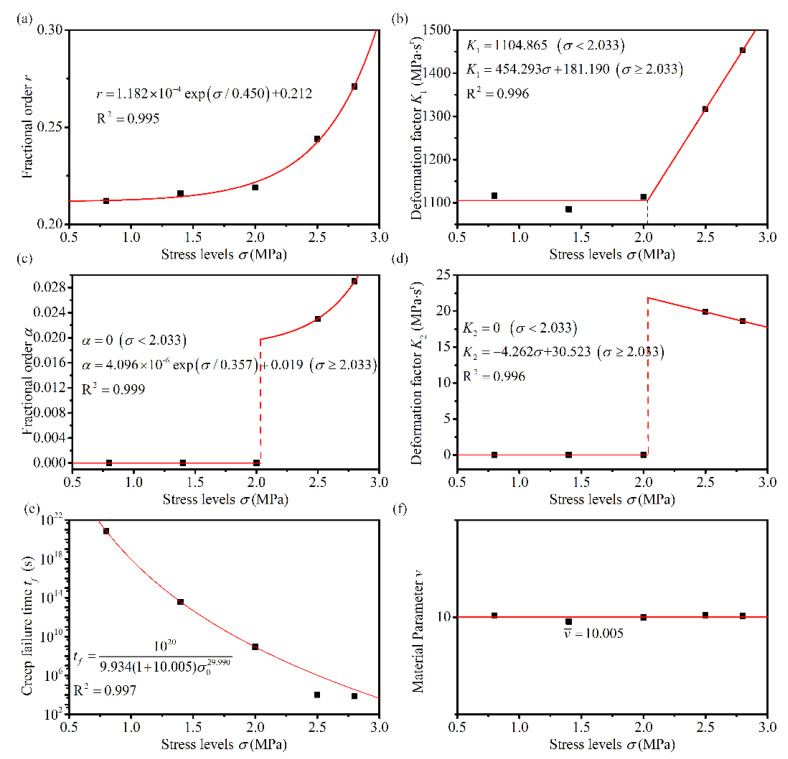
Variation of the proposed model parameters with stress levels at 10 °C (**a**) *r*, (**b**) *K*_1_, (**c**) *α*, (**d**) *K*_2_, (**e**) *t_f_*, and (**f**) *v*.

As can be seen from Table 4, several parameters in the fractional derivative creep damage model are related to both loading stress levels and temperatures. Figure 8, Figure 9 and Figure 10 illustrate the distribution of the parameters of various stress levels at different temperatures. Table 4 indicates that the material parameter *v* is essentially equal for different stresses at the same temperature; therefore, parameter *v* at a given temperature is considered the average of the parameter *v* at different stresses, as indicated in Figure 8f, Figure 9f and Figure 10f. When the asphalt mixtures were subjected to linear viscoelastic stress levels, the parameters *K*_1_ remained approximately constant, and parameters *α* and *K*_2_ were close to zero. Thus, the parameters *K*_1_, *α*, and *K*_2_ were considered constants in the range of the linear viscoelastic stresses. Under other stress levels, fractional orders *r* and *α* showed a good first-order exponential decay distribution regarding the stress levels, and the deformation factors, *K*_1_ and *K*_2_, showed a good linear distribution with the stress levels, as indicated in Figure 8, Figure 9 and Figure 10. The linear viscoelastic stress range could also be obtained using *K*_1_, as shown in Figure 9b and Figure 10b. The intersection point of the two straight lines, which described the relationship between *K*_1_ and the applied stress, was the point of demarcation between the linear viscoelastic range and the nonlinear viscoelastic range. When the stress level is less than the intersection point, the asphalt mixture is in the linear viscoelastic range and vice versa in the nonlinear viscoelastic range. The creep failure times at different stresses were fitted using Equation (12) to obtain the material parameters that describe the damage at different temperatures; the results are shown in Figure 8e, Figure 9e and Figure 10e.

**Figure 11:**
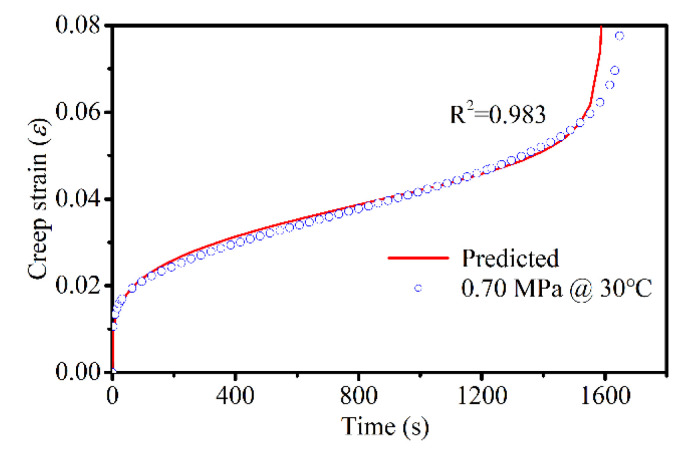
Comparison between the test data of the creep and predicted values at 30 °C and 0.7 MPa.

Based on the relationship between the model parameters and stresses given above, the creep curves could be predicted for any stress level at the three temperatures. Generally, the standard load was taken as 0.7 MPa when designing the pavement structure. Therefore, the creep behavior of the asphalt mixture was predicted and compared with the experimental data at 30 °C and 0.7 MPa, as shown in Figure 11. It was revealed that the predicted values were in good agreement with the experimental results.

### 4.4. Analysis of Damage Evolution

**Figure 12:**
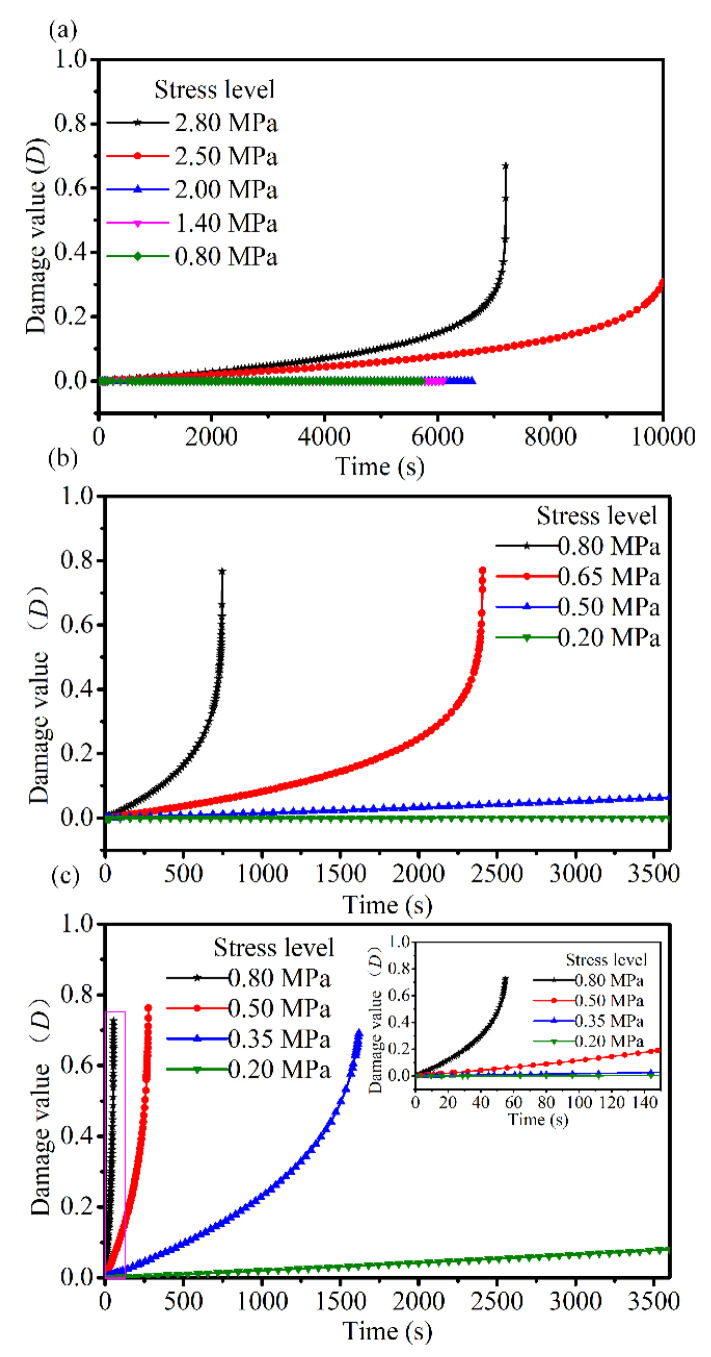
Variation curves of damage values with time under different compressive stress levels at (**a**) 10 °C, (**b**) 30 °C, and (**c**) 50 °C.

It is believed that the fractional derivative creep damage model provides a good description of the three-stage creep process in asphalt mixtures. Once the parameters of the proposed constitutive model are identified, the proposed damage evolution model can be applied to calculate the damage value *D*. The damage variation curves with a loading time at various stresses and temperatures are shown in Figure 12.

As indicated in Figure 12, at a given temperature, under higher stresses, the damage value D increases in an approximately linear fashion during the steady stage and rapidly during the tertiary stage. In contrast, at lower stresses, the damage value *D* remains at virtually zero throughout the creep process. Regardless of the applied stresses, the asphalt mixture exhibited negligible damage during the primary stage [16,34]. This is because, in the initial stages of loading, the asphalt mixture is compacted, which makes the mixture structurally hardened, at which point no damage occurs to the asphalt mixture. On a macroscopic scale, asphalt mixtures show a decrease in the void fraction and an increase in the resistance to deformation; this indicates creep hardening. Over time, the asphalt mixture structure continues to stabilize, and the flow of the asphalt mortar tends to slow down. However, the asphalt mortar and coarse aggregate interface are gradually damaged and develop into microcracks. Macroscopically, the asphalt mixture exhibits a stable void ratio and constant strain rate. As the asphalt mortar flow deformation continues to accumulate, microcracks coalesce to form macro cracks, the mineral skeleton of the asphalt mixture begins to destabilize, and the asphalt mixture enters an accelerated creep phase. Macroscopically, it is characterized by cracks in the asphalt mixture and a rapid reduction in load-bearing capacity until the mixture breaks down in the creep phase; in general, damage to the asphalt mixture is initiated in the steady stage and developed in the tertiary stage, which is consistent with the works reported by Zeng et al. [16] and Al-rub et al. [34]. The experimental data and model damage analysis show the feasability of the damage evolution proposed in this study.

Equation (11) considers only the time dependence of the damage evolution, assuming that the damage only develops over time; it does not consider the relationship between the damage evolution and the relevant mechanical quantities such as stress or strain. Equations (11) and (16) show that each moment t corresponds to a damage value and a creep strain value. Therefore, through creep tests, the relationship curve between the damage value and strain can be established for different stress levels. At different stress levels, the damage values corresponding to the strain values at each moment of the decelerated stage are small and essentially close to zero. The strain caused by the damage (damage strain, *ε_d_*) is obtained by subtracting the strain in the decelerated stage from the total creep strain in the tests. According to Equation (16), *ε_d_* can be expressed as:

(17) $$\varepsilon_{d}\left(t\right)=\varepsilon \left(t\right)-\frac{\sigma_{0}t^{r}}{K_{1}}.$$

**Figure 13:**
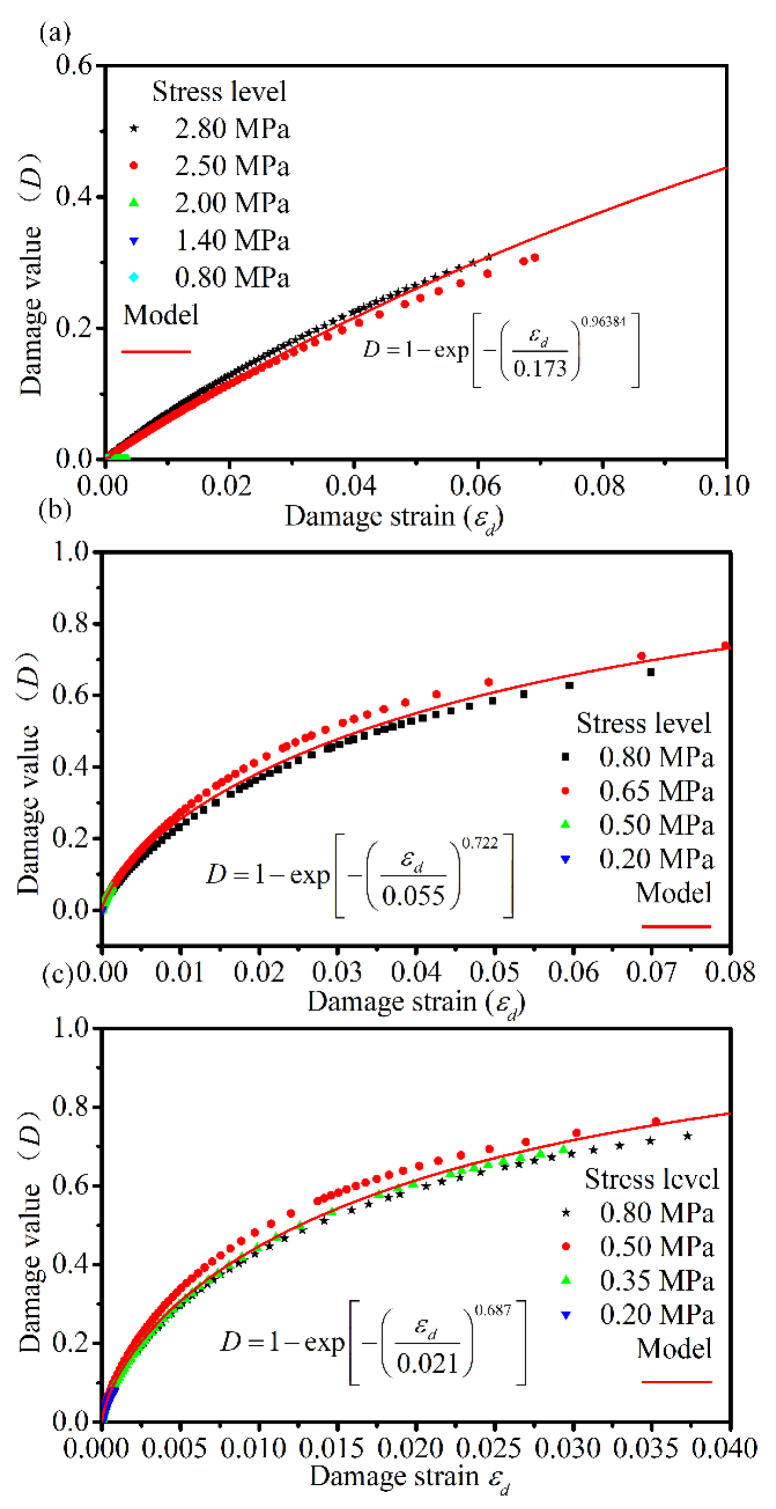
The relation curve between creep damage value and damage strain of asphalt mixture under different stress levels at (**a**) 10 °C, (**b**) 30 °C, and (**c**) 50 °C.

Based on the time dependence of the damage evolution and damage values obtained from the creep experiments, the relationship curves between the damage value *D* and *ε_d_* for different stress levels at different temperatures is established in this study, as shown in Figure 13. 

Figure 13 shows that the relationship curves between the creep *D* and the *ε_d_* of the asphalt mixture at different stress levels overlap. Therefore, a unified damage evolution model between *D* and *ε_d_* can be developed, where the damage parameters in the damage evolution relationship are only material- and temperature-dependent, independent of the magnitude of the stress levels. Katsuki et al. [4] used the Weibull distribution function to describe the variation in the damage with the strain, and verified the feasibility of the model using the experimental computed tomography images from Tashman at al. [35]. Therefore, in this study, the Weibull distribution function is used to describe the evolution of the damage with *ε_d_*, and the model expression is shown in Equation (18). By adopting the proposed damage model (Equation (18)) to fit the experimental data under different levels of stress for different temperatures in Figure 13, the damage model curve shown in Figure 13 is obtained. The parameters are listed in Table 5. The parameters of the correlation coefficient R^2^ are greater than 0.992, which shows that the Weibull distribution function can be used to describe the damage evolution and demonstrate the feasibility of establishing a unified damage evolution model for different stress levels. Statistically speaking, the microelement strength of randomly distributed microdefects within the material obeys the Weibull distribution under *ε_d_*:

(18) $$D=1-\exp\left[-{\left(\frac{\varepsilon_{d}}{p}\right)}^{q}\right],$$

where *p* and *q* are temperature-dependent material parameters, respectively.

The nonlinear damage constitutive model proposed in this study has the advantages of fewer parameters and a clear physical meaning of parameters relative to the creep model proposed by Zeng et al. [16]. Relative to the model proposed by Zhang et al. [19], there is no need to assume that there is critical stress. Additionally, relative to other damage models [16,17,19], this study decouples the damage strain from the creep strain and establishes a unified damage evolution model for different stress levels, whose parameters are only related to material properties and temperature, and are independent of the magnitude of the applied stress.

## 5. Conclusions

A fractional viscoelastic creep damage model was proposed to characterize the creep process of asphalt mixtures at different stress levels and temperatures based on the fact that the asphalt mixtures exhibited different deformation mechanisms throughout the entire creep process at different stress levels. The following conclusions were drawn:

(1) The compressive creep tests showed that the creep processes of asphalt mixtures at three different temperatures exhibited a consolidation effect at low stress levels and a tertiary stage at high stress levels. Based on the compressive creep tests, this study considered that the creep hardening and creep damage deterioration mechanisms were at work throughout the creep process. A nonlinear fractional creep damage model was proposed by combining the two models. The results of the compression creep tests were in good agreement with the proposed creep damage model, which confirmed the effectiveness of the model in describing the three-stage creep process of the asphalt mixture at different stress levels and temperatures.
(2) The parameters of the proposed model were explicit in a physical sense, and relationships between the parameters and the applied stresses were established. The creep consolidation stress range could be obtained through the relationship between *K*_1_ and the applied stresses.
(3) The damage value was negligible in the primary stage; it increased in an approximately linear fashion in the steady stage and rapidly during the tertiary stage. The damage was initiated in the steady stage and developed in the tertiary stage. The damage value remained at virtually zero throughout the creep process at low stresses; however, it increased rapidly with the increasing stress.
(4) Based on the statistical quantification of the asphalt mixture damage evolution, a unified creep damage evolution model, which represented the relationship between the damage evolution and damage strain, was established. The damage parameters in this damage evolution relationship were related to the material properties and temperature and were independent of the magnitude of the stress levels. The model described well the asphalt mixture damage evolution process, and the damage obeyed the Weibull distribution.

It should be noted that only the relationship between the model parameters and applied stresses was established in the work. In order to generalize the proposed nonlinear damage constitutive model, it is also necessary to provide a way to construct a relationship between the model parameters, temperature, and the applied stress. The establishment of the relationship between the three will be considered in future studies.

## Figures and Tables

**Table 1:** Performances of asphalt binder.

Type	Penetration (25 °C, 0.1 mm)	Ductility (15 °C, cm)	Softening Point (°C)	Brinell Viscosity (60 °C)	Elastic Recovery (%)
Virgin binder	70.1	60	47	285	10
Requirements	60–80	≥40	≥45	≥160	-
Specification	T0604 2011	T0605 2011	T0606 2011	T0625 2011	T0662 2000

**Table 2:** Performances of basalt aggregates.

Type	Crushing Value (%)	Los Angeles Abrasion Value (%)	Needle and Flaky Particle Content (%)	Water Absorption Rate (%)	Apparent Density (g/cm^3^)
Basalt aggregates	13.5	14.2	9.8	0.4	2.93
Requirements	≤26	≤28	≤15	≤2.0	>2.6
Specification	T0316 2005	T0317 2005	T0312 2005	T0307 2005	T0304 2005

**Table 3:** Creep experiment conditions of asphalt mixtures.

Testing Temperature/°C	Stress Levels /MPa
10	0.8, 1.4, 2.0, 2.5, 2.8
30	0.2, 0.5, 0.65, 0.8, 0.7 (validation)
50	0.2, 0.35, 0.5, 0.8

**Table 4:** The parameters of the proposed constitutive models.

Testing Tmperature /°C	Stress /MPa	*r*	*K*_1_/MPa·s^r^	*α*	*K*_2_/MPa·s*^α^*	*t_f_* /s	*v*	R^2^
10	2.80	0.271	1453.211	0.029	18.589	7350.634	10.072	0.999
	2.50	0.244	1316.923	0.023	19.8676	10,172.405	10.103	0.998
	2.00	0.219	1113.468	3.849 × 10^−6^	4.027 × 10^−4^	8.577 × 10^8^	9.987	0.999
	1.40	0.216	1084.970	0	1.407 × 10^−7^	3.792 × 10^13^	9.779	0.999
	0.80	0.212	1116.156	0	7.878 × 10^−12^	7.661 × 10^18^	10.085	0.999
30	0.80	0.225	141.968	0.396	310.243	746.492	5.174	0.999
	0.65	0.165	125.024	0.350	352.432	2407.442	5.267	0.999
	0.50	0.131	104.158	0.326	386.826	10,525.528	5.361	0.998
	0.20	0.080	73.064	0	2.196	1.8014 × 10^6^	5.248	0.999
50	0.80	0.254	144.683	0.517	455.567	55.552	2.558	0.999
	0.50	0.147	106.803	0.441	542.917	278.371	2.599	0.999
	0.35	0.115	96.704	0.419	587.824	1643.618	2.583	0.999
	0.20	0.080	79.432	0.411	624.145	13,912.083	2.560	0.998

**Table 5:** The parameters of damage evolution model.

Testing Temperature/°C	*p*	*q*	R^2^
10	0.173	0.964	0.992
30	0.055	0.722	0.995
50	0.021	0.687	0.995

## References

[1] Ahmed I., Thom N., Bilal Ahmed Zaidi S., Carvajal-Munoz J.S., Rahman T., Dawson A. (2021). Application of a novel linear-viscous approach to predict permanent deformation in simulative inverted pavements. Constr. Build. Mater..

[2] Doll B., Ozer H., Rivera-Perez J.J., Al-Qadi I.L., Lambros J. (2017). Investigation of viscoelastic fracture fields in asphalt mixtures using digital image correlation. Int J. Fract..

[3] Dong N., Wang D., Zhang S., Chen Z., Liang H., Ni F., Yu J., Yu H. (2021). Exploring creep and recovery behavior of hot mix asphalt field cores with multi-sequenced repeated load test. Constr. Build. Mater..

[4] Katsuki D., Gutierrez M. (2011). Viscoelastic damage model for asphalt concrete. Acta Geotech..

[5] Nguyen Q.T., Di Benedetto H., Nguyen Q.P., Hoang T.T.N., Bui V.P. (2021). Effect of time–temperature, strain level and cyclic loading on the complex Poisson’s ratio of asphalt mixtures. Constr. Build. Mater..

[6] Zbiciak A., Brzeziński K., Michalczyk R. (2017). Constitutive models of pavement asphaltic layers based on mixture compositions. J. Civ. Eng. Manag..

[7] Ghorbani B., Arulrajah A., Narsilio G., Horpibulsuk S. (2020). Experimental and ANN analysis of temperature effects on the permanent deformation properties of demolition wastes. Transp. Geotech..

[8] Alrashydah E.A.I., Abo-Qudais S.A. (2018). Modeling of creep compliance behavior in asphalt mixes using multiple regression and artificial neural networks. Constr. Build. Mater..

[9] Ghorbani B., Arulrajah A., Narsilio G., Horpibulsuk S., Win Bo M. (2021). Thermal and mechanical properties of demolition wastes in geothermal pavements by experimental and machine learning techniques. Constr. Build. Mater..

[10] Bai F., Yang X., Zeng G. (2017). Stochastic Viscoelastic–Viscoplastic Response of Asphalt Mixture under Uniaxial Compression. J. Eng. Mech..

[11] Gao D., Wang P., Li M., Luo W. (2015). Modelling of nonlinear viscoelastic creep behaviour of hot-mix asphalt. Constr. Build. Mater..

[12] Huang C., Wang F., Gao T., Gao D., Kachanov L.M. (2018). A New Viscoelastic Mechanics Model for the Creep Behaviour of Fibre Reinforced Asphalt Concrete. Frat. Integrita Strut..

[13] Lagos-Varas M., Movilla-Quesada D., Arenas J.P., Raposeiras A.C., Castro-Fresno D., Calzada-Pérez M.A., Vega-Zamanillo A., Maturana J. (2019). Study of the mechanical behavior of asphalt mixtures using fractional rheology to model their viscoelasticity. Constr. Build. Mater..

[14] Lagos-Varas M., Raposeiras A.C., Movilla-Quesada D., Arenas J.P., Castro-Fresno D., Muñoz-Cáceres O., Andres-Valeri V.C. (2020). Study of the permanent deformation of binders and asphalt mixtures using rheological models of fractional viscoelasticity. Constr. Build. Mater..

[15] Luo W., Li B., Zhang Y., Yin B., Dai J. (2020). A Creep Model of Asphalt Mixture Based on Variable Order Fractional Derivative. Appl. Sci..

[16] Zeng G., Yang X., Bai F., Gao H. (2014). Visco-elastoplastic damage constitutive model for compressed asphalt mastic. J. Cent. South. Univ..

[17] Zhang J., Li Z., Chu H., Lu J. (2019). A viscoelastic damage constitutive model for asphalt mixture under the cyclic loading. Constr. Build. Mater..

[18] Zhang J., Zhu C., Li X., Pei J., Chen J. (2017). Characterizing the three-stage rutting behavior of asphalt pavement with semi-rigid base by using UMAT in ABAQUS. Constr. Build. Mater..

[19] Zhang Y., Liu X., Yin B., Luo W. (2021). A Nonlinear Fractional Viscoelastic-Plastic Creep Model of Asphalt Mixture. Polymers.

[20] Luo W., Jazouli S., Vu-Khanh T. (2007). Modeling of Nonlinear Viscoelastic Creep of Polycarbonate. e-Polymers.

[21] Darabi M.K., Huang C.-W., Bazzaz M., Masad E.A., Little D.N. (2019). Characterization and validation of the nonlinear viscoelastic-viscoplastic with hardening-relaxation constitutive relationship for asphalt mixtures. Constr. Build. Mater..

[22] Im S., You T., Ban H., Kim Y.-R. (2017). Multiscale testing-analysis of asphaltic materials considering viscoelastic and viscoplastic deformation. Int. J. Pavement. Eng..

[23] Chang Kuo-Neng G., Meegoda Jay N. (1997). Micromechanical Simulation of Hot Mix Asphalt. J. Eng. Mech..

[24] Cheng Y., Li H., Li L., Zhang Y., Wang H., Bai Y. (2019). Viscoelastic Properties of Asphalt Mixtures with Different Modifiers at Different Temperatures Based on Static Creep Tests. Appl. Sci..

[25] Xu X., Cui Z. (2020). Investigation of a fractional derivative creep model of clay and its numerical implementation. Comput. Geotech..

[26] Gu L., Zhang W., Ma T., Qiu X., Xu J. (2021). Numerical Simulation of Viscoelastic Behavior of Asphalt Mixture Using Fractional Constitutive Model. J. Eng. Mech..

[27] Xu Y., Shan L., Tian S. (2019). Fractional Derivative Viscoelastic Response Model for Asphalt Binders. J. Mater. Civil. Eng..

[28] Celauro C., Fecarotti C., Pirrotta A., Collop A.C. (2012). Experimental validation of a fractional model for creep/recovery testing of asphalt mixtures. Constr. Build. Mater..

[29] Sun L., Zhu H., Zhu Y. (2013). Two-Stage Viscoelastic-Viscoplastic Damage Constitutive Model of Asphalt Mixtures. J. Mater. Civil. Eng..

[30] Koeller R.C. (1984). Applications of Fractional Calculus to the Theory of Viscoelasticity. J. Appl. Mech..

[31] Kachanov L.M. (1999). Rupture Time Under Creep Conditions. Int J. Fract..

[32] Lemaitre J. (1984). How to use damage mechanics. Nucl. Eng. Des..

[33] Luo W., Liang S., Zhang Y. (2020). Fractional Differential Constitutive Model for Dynamic Viscoelasticity of Asphalt Mixture. China J. Highw. Transp..

[34] Al-rub R.K.A., You T., Masad E.A., Little N. (2011). Mesomechanical modeling of the thermo-viscoelastic, thermo-viscoplastic, and thermo-viscodamage response of asphalt concrete. Int J. Eng. Sci..

[35] Tashman L., Masad E., Little D., Zbib H. (2005). A microstructure-based viscoplastic model for asphalt concrete. Int J. Plast..

